# Remote Control of Greenhouse Vegetable Production with Artificial Intelligence—Greenhouse Climate, Irrigation, and Crop Production

**DOI:** 10.3390/s19081807

**Published:** 2019-04-16

**Authors:** Silke Hemming, Feije de Zwart, Anne Elings, Isabella Righini, Anna Petropoulou

**Affiliations:** Wageningen University & Research, Business Unit Greenhouse Horticulture, 6708PB Wageningen, The Netherlands; feije.dezwart@wur.nl (F.d.Z.); anne.elings@wur.nl (A.E.); isabella.righini@wur.nl (I.R.); anna.petropoulou@wur.nl (A.P.)

**Keywords:** artificial intelligence, sensors, resource use efficiency, crop production, indoor farming

## Abstract

The global population is increasing rapidly, together with the demand for healthy fresh food. The greenhouse industry can play an important role, but encounters difficulties finding skilled staff to manage crop production. Artificial intelligence (AI) has reached breakthroughs in several areas, however, not yet in horticulture. An international competition on “autonomous greenhouses” aimed to combine horticultural expertise with AI to make breakthroughs in fresh food production with fewer resources. Five international teams, consisting of scientists, professionals, and students with different backgrounds in horticulture and AI, participated in a greenhouse growing experiment. Each team had a 96 m^2^ modern greenhouse compartment to grow a cucumber crop remotely during a 4-month-period. Each compartment was equipped with standard actuators (heating, ventilation, screening, lighting, fogging, CO_2_ supply, water and nutrient supply). Control setpoints were remotely determined by teams using their own AI algorithms. Actuators were operated by a process computer. Different sensors continuously collected measurements. Setpoints and measurements were exchanged via a digital interface. Achievements in AI-controlled compartments were compared with a manually operated reference. Detailed results on cucumber yield, resource use, and net profit obtained by teams are explained in this paper. We can conclude that in general AI performed well in controlling a greenhouse. One team outperformed the manually-grown reference.

## 1. Introduction

The global population is increasing rapidly together with the demand for healthy fresh food [1]. The greenhouse industry can play an important role providing fresh food, such as fruits and vegetables being high in vitamins and minerals. Greenhouses allow a high crop production per area combined with a high water use efficiency per unit of produce [2]. Worldwide, the area of greenhouse production is increasing [3]. However, the greenhouse industry encounters difficulties finding enough skilled labor to manage crop production [4]. A crop manager must have a high level of knowledge and experience in order to control crop growth. As farms become larger, monitoring all details of the various greenhouse compartments becomes more demanding. Moreover, resources (water, fossil energy) are becoming scarcer, which causes an urgent need for maximum resource efficiency.

A greenhouse protects the crop from outside influences, such as rain, wind, low temperatures, or pests. A modern high-tech greenhouse is equipped with active control of actuators (e.g., heating, lighting, irrigation) in order to create a favorable growing climate. Of course, this comes at the cost of resource consumption (e.g., fuel, electricity, water). A grower determines the climate and irrigation strategy and defines the setpoints for all climate and irrigation parameters. Actuators are operated based on the setpoints, and sensors give feedback on measured data for the control loop. Automated greenhouse climate control algorithms have already been developed decades ago [5,6,7,8,9,10,11,12]. Today, modern high-tech greenhouses are equipped with process computers, which are able to control greenhouse actuators based on the setpoints manually set by the grower.

In order to add more automated control, various greenhouse climate and crop models have been developed. An overview of today’s greenhouse climate models is given in a previous study [13]. An overview of greenhouse crop models and modelling approaches are given in other studies [14,15]. Dynamic greenhouse climate models and dynamic crop models have been used to determine setpoints automatically and take over the decision of the grower. If climate and crop simulation models [15,16] are combined and connected to the sensors and actuators of a greenhouse, greenhouse climate and crop growth can be controlled by automated algorithms. Such experiments have been conducted successfully with tomato [17] and sweet pepper in The Netherlands [18,19]. In this experiment, outside weather conditions and weather forecasts were used for climate simulations. Crop growth simulations were carried out along with the cropping cycle to predict further crop growth and development for different sets of setpoints. The optimum set was then applied in the greenhouse automatically. The computations were repeated every day, and in this way, crops were grown with an optimum control strategy. Other experiments with tomato have previously been conducted [20].

Another way to take over parts of the decisions of a grower is to use machine learning algorithms for greenhouse climate control [21]. Diverse methods have been applied in research, such as K-algorithms [22], Bayesian networks [23], support vector machines regression [24,25,26], neural networks [27,28,29,30,31,32,33,34], reinforcement learning [35], or genetic algorithms [36,37,38]. However, to our knowledge machine learning has not been used yet to control climate and irrigation and make crop management decisions for growing a greenhouse crop autonomously during a longer period with yield levels comparable to commercial practice.

On the other hand, the use of artificial intelligence (AI) has reached major breakthroughs in several areas of daily life and society, such as medical applications [39], autonomous cars [40], or robotics [41]. AI algorithms have been shown to outperform humans in complex decisions, e.g., checkers [42], chess [43], and go [44]. It is obvious to use AI also for agricultural purposes [45].

In order to combine the use of modern AI algorithms and greenhouse climate, irrigation, and crop growth control, in 2018 an international challenge on “autonomous greenhouses” was conducted at the high-tech research greenhouses of Wageningen University and Research in cooperation with five multi-disciplinary international teams. The challenge aimed at combining horticultural expertise with AI to make breakthroughs in fresh food production with fewer resources. The experiment was set-up with the goal of benchmarking the use of state-of-the-art AI algorithms for cucumber production. In the experiment existing commercial greenhouse equipment (actuators), standard sensors for measurement and control, and a standard commercial process computer were combined with the latest AI technology in order to maximize net profit and minimize resource use, while controlling greenhouse crop growing remotely. The goal of this paper is to describe the results obtained by teams concerning net profit and resource use, to analyze differences in climate and crop growing strategies used, and to investigate which lessons can be learned from the results for the future, in terms of optimizing crop yields and net profit.

## 2. Materials and Methods

### 2.1. Greenhouse Compartments and Actuators

**Figure 1:**
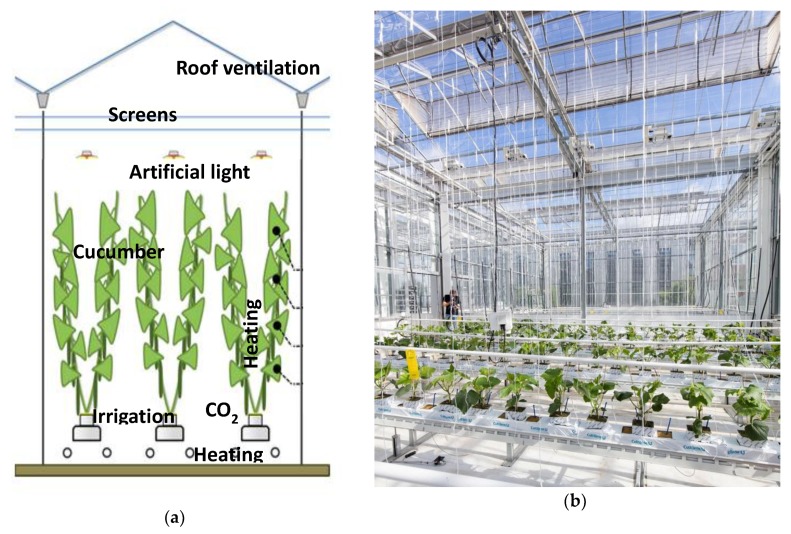
Greenhouse experimental compartments, 96 m^2^ ground floor (76.8 m^2^ crop-growing area) equipped with different actuators. (**a**) Scheme of compartment with crop and actuators: roof ventilation, two screens, artificial light, irrigation system, CO_2_ supply, two heating systems. (**b**) Picture of one compartment with the young crop after the transplant.

Six identical greenhouse compartments were available for the cucumber growing experiment. Each compartment was equipped with standard actuators, also available in commercial high-tech greenhouses (Figure 1). Two pipe heating systems, a rail pipe heating on the floor, and a pipe heating on crop height (peak capacity 180 and 30 W/m^2^ respectively), were available, both controllable by different setpoints. Continuous roof ventilation (ventilation area of 0.3 m^2^ opening per m^2^ greenhouse, equipped with anti-thrips netting), two types of inside moveable screens (LUXOUS 1547 D FR energy screen and OBSCURA 9950 FR W light blocking screen, Ludvig Svenssion, Sweden), a high-pressure-sodium artificial lighting system (capacity of 187 μmol/m^2^/s), a fogging system (maximum capacity of 330 g/m^2^/h), and CO_2_ supply (maximum capacity 15 g/m^2^/h) were available. Plants were grown in rockwool substrate cubes and placed on rockwool substrate slabs; the plant-substrate system was then located on hanging gutters. Irrigation water and nutrients were supplied with drippers operated by a valve. The surplus of the nutrient solution (drain) was recollected in the hanging gutter in a closed loop system.

### 2.2. Sensors and Remote Control

**Figure 2:**
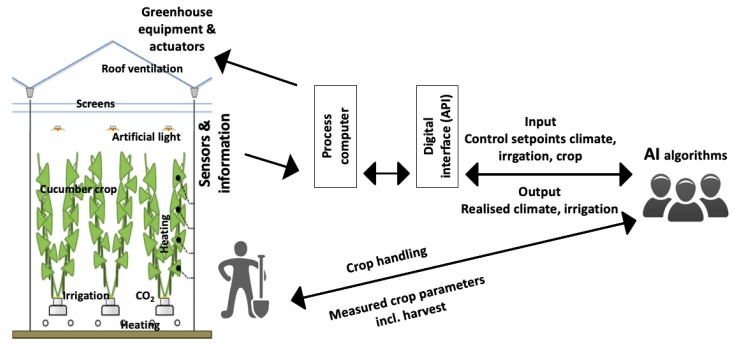
Scheme of data exchange from the teams and their AI algorithm via a digital interface (REST API) towards the process computer and the greenhouse actuators and data from sensors via the same way back, data exchange between teams and workers on crop handling, and measured crop parameters.

Five teams (Sonoma, iGrow, deep_greens, The Croperators, AiCU) were able to control the operation of all actuators remotely based on their own AI algorithm. A sixth greenhouse compartment was controlled by Dutch growers and served as a reference (growers = reference). Competing teams used their own AI algorithms to determine the climate and irrigation control setpoints, such as minimum rail pipe temperature (°C), minimum crop pipe temperature (°C), heating temperature (°C), ventilation temperature (°C), minimum ventilation opening (%), humidity deficit setpoint (g/m^3^), energy screen position (0–100%), blackout screen position (0–100%), artificial illumination (0% or 100%), CO_2_ concentration (ppm), and time between last and next irrigation turn (min). Setpoints were sent via a digital interface (LetsGrow.com) to a central climate process computer (IISI, Hoogendoorn, The Netherlands), which then operated the actuators accordingly (Figure 2). A nutrient solution for fertigation was prepared by a central fertigation computer and then stored in a buffer tank per compartment before being provided to the crop with drippers. The composition, concentration (EC), and pH of the nutrient solution was determined by the teams. Based on detailed chemical analysis of the drain water, provided every fortnight, the teams could send requests to change the composition, EC, and pH of the nutrient solution.

Standard sensors continuously measured data, such as cumulative outside global radiation (J/cm^2^/d), outside photosynthetically active radiation PAR (μmol/m^2^/s), air temperature outside (°C), outside relative humidity (%), wind speed (m/s), outside global radiation forecast (W/m^2^), outside air temperature forecast (°C), outside relative humidity forecast (%), wind speed forecast (m/s), air temperature inside (°C), air humidity deficit inside (g/m^3^), heating pipe temperature (°C), heating power used (W/m^2^) for both heating systems, lamp status (on/off), CO_2_ dosage (on/off), screen position (%) of both screens, irrigation supply (l/m^2^), drain (l/m^2^), drain EC (dS/m), and drain pH (−). The following data was calculated from the measured data: inside PAR sum (mol/m^2^), heating energy used (kWh/m^2^), electricity used (kWh/m^2^), CO_2_ dosage (kg/m^2^), water consumption (l/m^2^), and was provided to the teams as well. Measurements and calculations were sent back to the teams via a digital interface (Figure 2). Both, setpoints for control of actuators and measurements were exchanged at a 5-min-interval.

Teams were allowed to install additional sensors at the start of the experiment. They chose different types of sensors, such as RGB cameras, thermal cameras, sensors for net radiation, root zone sensors, crop and substrate weight, stem diameter, crop sap flow meters, and wireless temperature-humidity-light sensor networks. One team chose to rely on the standard greenhouse sensors only (iGrow).

### 2.3. Crop and Crop Parameters

**Figure A1:**
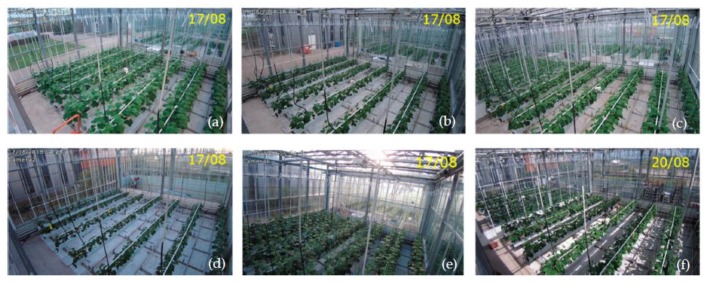
Young cucumber crop planted in the six greenhouse compartments, pictures of different stem densities chosen by teams: (**a**) iGrow; (**b**) deep_greens; (**c**) AiCU; (**d**) Sonoma; (**e**) Reference; (**f**) The Croperators.

**Figure A2:**
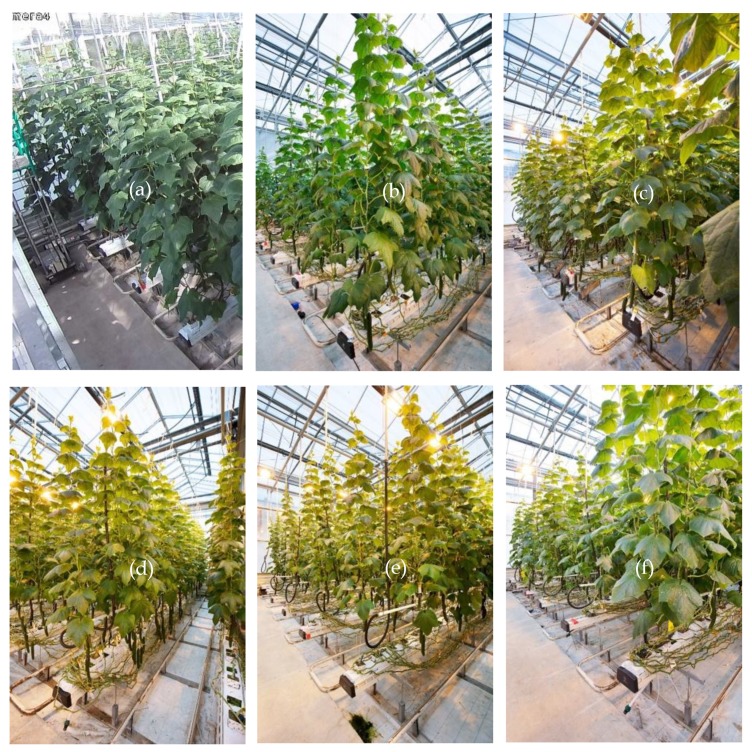
Cucumber crop after 1 month, pictures of different compartments of teams: (**a**) iGrow; (**b**) deep_greens; (**c**) AiCU; (**d**) Sonoma; (**e**) Reference; (**f**) The Croperators.

**Figure A3:**
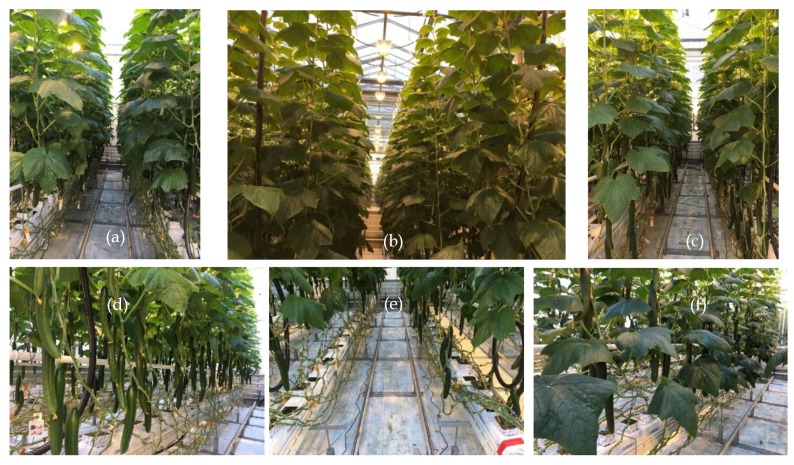
Cucumber crop after 4 months, pictures of different compartments of teams: (**a**) iGrow; (**b**) deep_greens; (**c**) AiCU; (**d**) Sonoma; (**e**) Reference; (**f**) The Croperators.

**Figure A4:**
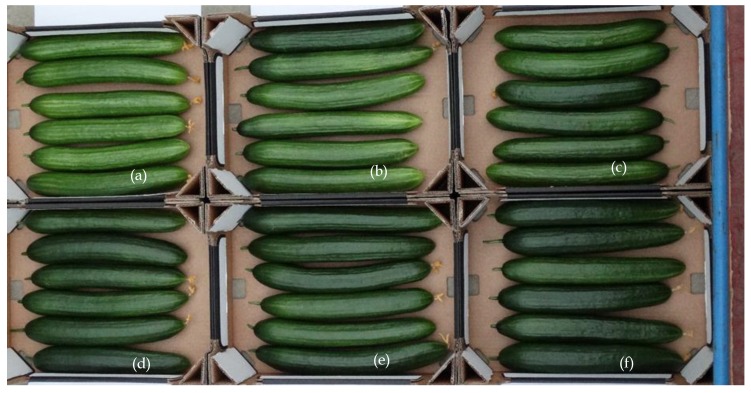
Cucumbers harvested, pictures of fruits from different compartments of teams: (**a**) The Croperators; (**b**) Sonoma; (**c**) AiCU; (**d**) Reference; (**e**) deep_greens; (**f**) iGrow.

Cucumbers seedlings cv. “Hi-Power” were sown on 20 July 2018, in rockwool cubes and were transplanted to the greenhouse compartments on 14 August 2018, at the start of the experiment. The crop was grown in a high-wire growing system. Plant density and stem density had to be chosen by the teams before the start, resulting in values between 2.6 and 3.6 stems/m^2^ (iGrow, 2.6 stems/m^2^; deep_greens, 2.6 stems/m^2^; AiCU, 3.6 stems/m^2^; Sonoma, 3.3 stems/m^2^; The Croperators, 3.2 stems/m^2^). The reference was 2.5 stems/m^2^. The first harvest was on 6 September 2018, and the last harvest was scheduled for all teams on 7 December 2018. Based on this last harvest date, the date of topping (removal of head of the crop) had to be chosen by the teams and differed from 19–28 November 2018. The reference was topped on 9 November 2018. Crop development and harvest is shown in Figure A1, Figure A2, Figure A3 and Figure A4.

Teams sent weekly instruction for fruit and leaf pruning in the top of the canopy to the greenhouse workers. Fruit pruning strategies ranged from a stable procedure of 50% fruit removal for the whole cropping period to a more variable strategy of removing alternately 50% and 67% of the fruits. With respect to leaf pruning, the majority of the teams decided for on pruning (0%) or on pruning a small fraction of leaves (33%). One team used a deviating strategy of removing 50% of the leaves throughout the whole cropping. As a standard procedure applied to all crops, greenhouse workers removed leaves below last harvested fruits. Three harvest quality categories were distinguished (A: >375 g and no defects, B: 300–374 g or defects e.g., shape, color, others, C: <300 g per fruit). Harvest data such as number and weight of fruits (#/m^2^ and kg/m^2^ per quality category A–C) were measured manually by the workers. Crop related parameters such as stem elongation (cm per week), fruit growth period (d per fruit), leaf formation rate (# per stem per week), and cumulative number of leaves (# per stem) were also measured. Instructions by teams and data measured by workers were exchanged via the digital interface (Figure 2).

### 2.4. AI Algorithms

Each competing team developed their own AI algorithms, which varied between supervised, unsupervised, and reinforcement machine learning (Dynamic Regression, Deep Reinforcement Learning DRL, Deep Deterministic Policy Gradient DDPG, Generative Adversarial Networks GAN, Convolutional Neural Networks CNN, Recurrent Neural Networks RNN).

In order to use AI techniques, training data is essential. Since training data with a wide variation for the described application are scarce, an artificial training data set was created. The use of artificial training data sets has been shown to be very useful in other applications earlier [46]. In this experiment artificial training data was created using the broadly validated dynamic greenhouse climate model KASPRO [16] and the cucumber crop model INTKAM [15] that was modified for a high-wire cucumber crop. The artificial dataset was provided to the teams before the start of the experiment.

### 2.5. Performance Criteria

Teams’ performance was evaluated based on three criteria.

Sustainability: 20% of the total score of a team was given for sustainability. The following aspects were calculated based on measured data: Energy use efficiency (MJ/kg cucumber), CO_2_ dosage (kg/kg cucumber), water use efficiency (m^3^/kg cucumber), pesticide usage as registered (mL/cucumber).

Net profit: 50% of the total score of a team was given for the net profit. Net profit was calculated based on the following obtained data: number of fruits harvested x price per fruit and category. The prices varied per week during the cultivation period and were determined by a jury at the start of the experiment, and reflected an average seasonal trend. The prices varied between €0.30 and €0.40 per fruit (class A). B-class fruits had a 15% lower value, and C-class fruits had no value. The prices were revealed on a weekly basis during the growing cycle in order to mimic price uncertainty, typical for agricultural products in practice. Also, the initial costs for the young plants (costs of a young plant x number of young plants placed in the compartment) and costs of the substrate were considered. This way, the teams had to weigh the faster initial growth of a high stem density crop against the higher initial costs. Other greenhouse equipment used was identical, and therefore not considered in the calculation of the net profit. The fact that capital costs equal for all teams were left out of consideration explains the high values of net profit shown in the results. Resource use of electricity, heating, CO_2_, water, nutrients, chemical and biological pesticides, and labor were measured during the experiment per greenhouse compartment, and assigned to the teams. Multiplied with the given price, costs were calculated and communicated with teams weekly during the ongoing experiment.

AI algorithm: 30% of the total score of teams was given by a jury based on novelty of the AI algorithm with respect to the overall scientific community, novelty with respect to application on the horticultural domain (novelty), capacity to operate autonomously at a distance without manual interventions (functionality), capacity to operate without too many additional sensors or information (robustness), and easiness of implementation on a large scale (scalability).

### 2.6. Analysis and Interpretation of Results

The AI-based operation of the different greenhouse compartments by different teams resulted in different cropping, climate, and irrigation strategies, and different yields and resource use efficiencies. In order to properly analyze and compare the different approaches, a combination of a dynamic greenhouse climate model KASPRO [16] and a cucumber crop simulation model INTKAM [15], which was modified for a high-wire cucumber crop, was used. The combined model assumes adequate supply of water and nutrients and does not simulate the presence and effects of pests and diseases.

The KASPRO model computes the greenhouse climate as a function of outside weather conditions and greenhouse climate control settings. The model processes these settings by a control algorithm comparable to the ones used. The analysis was by commercial greenhouse climate computers. The model takes full account of the limitations of real greenhouses, which means, for instance, that a CO_2_ dosing setpoint of 800 ppm is simply not met in sunny periods when the vents are wide open to carry off the heat excess. This is caused by the limitations in maximal supply rate, just like in real greenhouses.

The greenhouse climate, as computed by KASPRO [16], is then fed to INTKAM [15], which computes the daily gross photosynthesis from the sum of hourly photosynthesis-rates. The hourly values are the result of light-intensity, temperature, CO_2_-concentration, and relative air humidity in combination with the dynamically-simulated crop architecture (in particular leaf area index). After subtracting maintenance costs, the daily amount of assimilates is partitioned over the growing organs (roots, stem, leaves, and fruits) on the basis of their relative potential growth rates. Next, dry matter fraction and fresh organ weights are computed, and finally the harvest moment of individual fruits is determined on the basis of, amongst others, fruit weight [15].

With the availability of these models, the contribution of the variation seen in the control strategies of the different teams in the final production could be determined. The variations applied referred to both the observed cropping and climate strategies to interpret and understand the results of the different AI-based operations and identify which additional improvements could have been made.

In a first step, the combined model was used to calculate the cucumber yield of each of the compartments, while using the actually applied crop density, fruit and leaf pruning strategy, and the realized lighting and climate (temperature and CO_2_) setpoints in that compartment as model inputs. The calculated model output was the predicted fresh yield (kg/m^2^, #fruits/m^2^) per greenhouse compartment, which could then be compared with the realized yield in the same greenhouse compartment to validate the models.

In a next step, for each greenhouse compartment, model calculations were carried out applying the *cropping strategy* of *other* teams or the reference in order to predict the changes in yield while maintaining the original lighting and climate strategy. In another step, model calculations were carried out for each greenhouse compartment, maintaining the original cropping and climate strategy but applying the *lighting strategy* of the *other* teams. In another step, calculations were made for each greenhouse compartment applying the *climate strategy* (*CO*_2_) of each of the *other* teams, while maintaining the original cropping and lighting strategy. For joint comprehensibility, interactions of cropping, lighting, and climate strategies were not calculated. The simulations of the swapping strategies represent the yield retrieved prior to topping, to eliminate the effect of early topping dates selected by some of the teams.

## 3. Results

**Figure 3:**
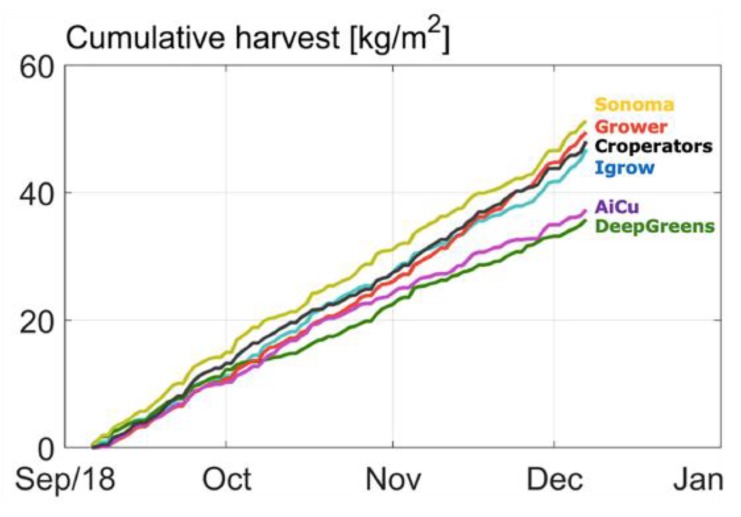
Cumulative cucumber production of different teams.

**Figure 4:**
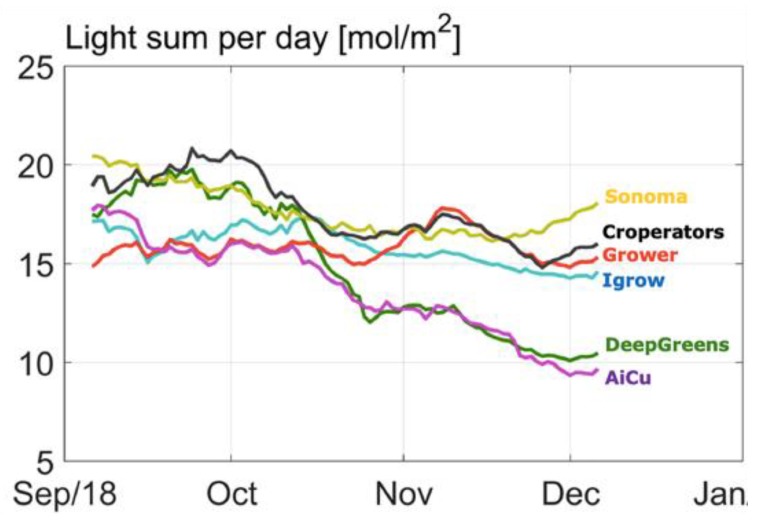
Course of total daily light integral in mol/m^2^ inside the greenhouse just above the crop, composed of both artificial light and natural light, realized by different teams.

**Figure 5:**
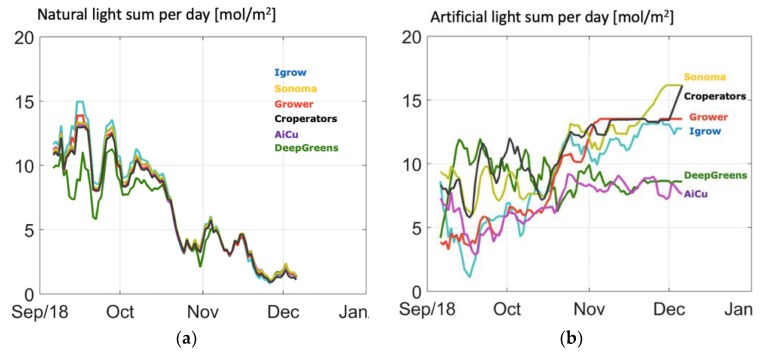
Course of daily light integral of (**a**) natural light and (**b**) artificial light only inside the greenhouse, just above the crop realized by different teams.

**Figure 6:**
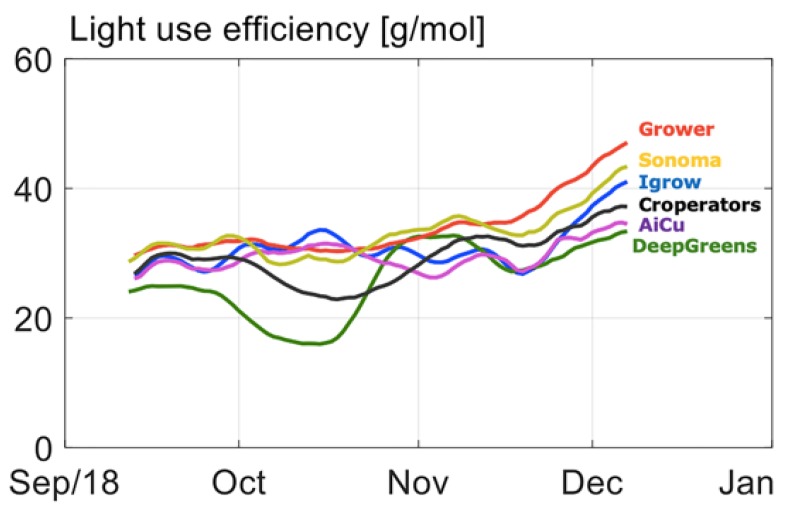
Course of light use efficiency in g cucumber per mol total daily light integral in different compartments.

In Figure 3, the cumulative cucumber production per team in the different greenhouse compartments during the experimental period is given. From the beginning, one team (Sonoma) had the highest production and was able to continue this, as is shown by the highest curve slope. This team obtained this harvest with a high daily light integral (Figure 4), as they assumed that with a higher daily light integral a greater harvest could be obtained. Therefore, team Sonoma focused its AI algorithms on this particular aspect. The algorithm allowed them to obtain a high daily light integral by maximizing the amount of artificial light (Figure 5b), while optimizing other defining factors, such as temperature and CO_2_. Hence, they realized the highest yield and they were also able to maintain high light use efficiency of the crop for a long period (Figure 6).

**Figure 7:**
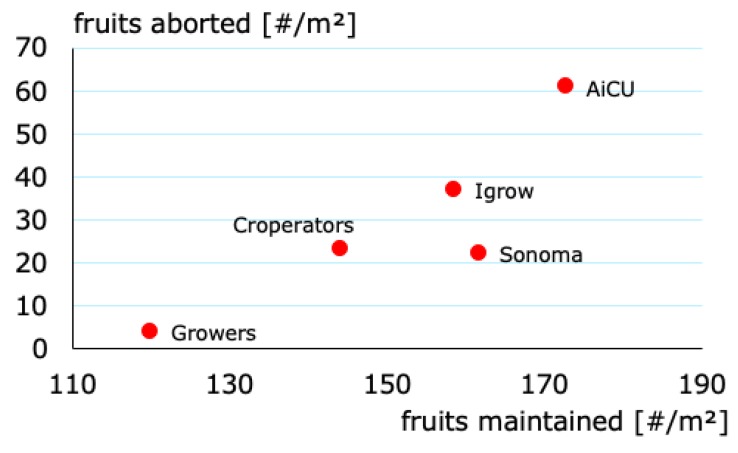
Number of aborted fruits and number of maintained fruits based on chosen fruit pruning strategy per team during the cucumber growing experiment.

Another team (The Croperators) increased the daily light integral after a short period at the beginning of October (Figure 4 and Figure 5b), however, this did not lead to a higher light use efficiency in October (Figure 6), since at the same time they maintained a low CO_2_ concentration. In addition, they opted for a crop pruning strategy that resulted in insufficient assimilates to sustain the growth of all fruits, and is probably the cause of approximately 30% aborted fruits (Figure 7). These results show that a high daily light integral and a high CO_2_ concentration are important production factors, which is related to their effect on the photosynthesis rate [47] for cucumber production, together with balanced crop management (stem density and pruning strategy).

Growers (reference) started with a relatively low harvest. They allowed lower daily light integrals at the beginning (Figure 4), and therefore only used low levels of artificial light (Figure 5b), with the philosophy that this approach would prepare the crop better for the approaching autumn season. They were able to balance supply of, and demand for, assimilates with their crop pruning strategy, which resulted in the lowest amount of aborted fruits (Figure 7) and a high light use efficiency (Figure 6). In fact, the manual growers were able to realize the highest light use efficiency during almost the whole cropping cycle. Minimizing fruit abortion is an important objective for cucumber growers, and this strategy was clearly and successfully applied by the manual growers.

**Figure 8:**
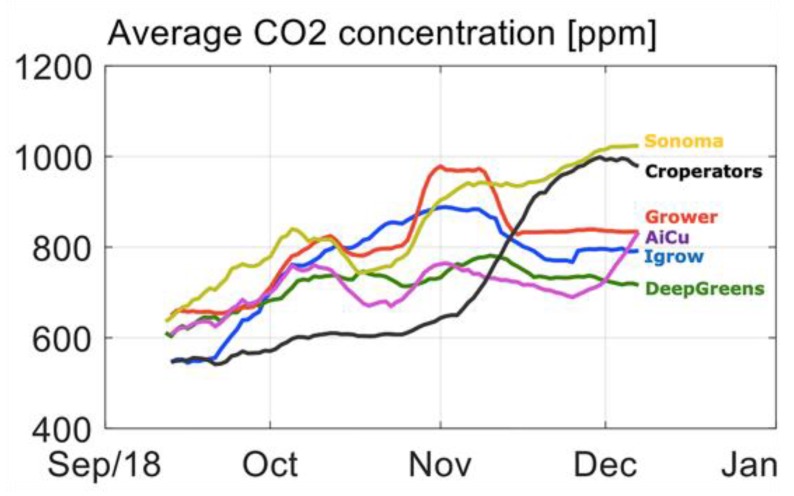
Course of CO_2_ concentration in ppm in different compartments during the cucumber experiment.

**Figure 9:**
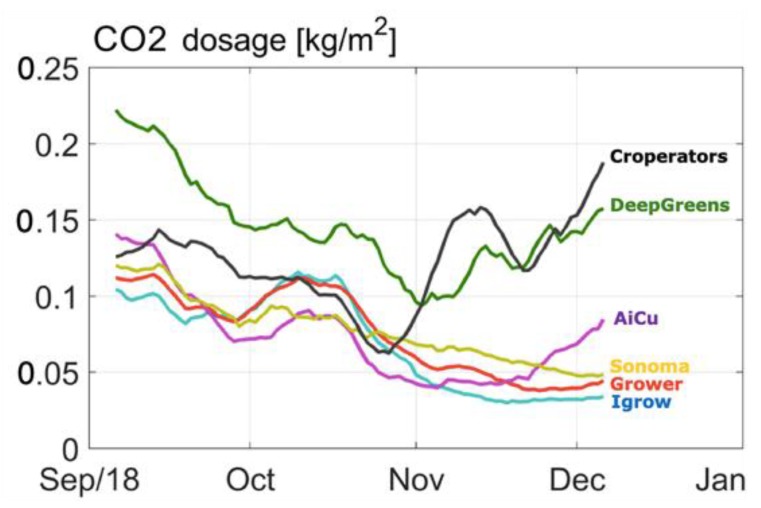
Course of CO_2_ dosage in kg/m^2^ in different compartments during the cucumber experiment.

Figure 8 and Figure 9 show the CO_2_ concentration and the CO_2_ dosage realized per team, respectively. All teams started with relatively low CO_2_ concentration, due to loss of CO_2_ due to high ventilation rates. Most teams increased CO_2_ concentration from mid-October onwards. From mid-November towards the end of the experiment, most teams lowered the CO_2_ dosage. Team Sonoma increased the CO_2_ concentration continuously during the total cropping period. The Croperators suddenly doubled the dosage and concentration towards the end of the crop (Figure 9) and were able to catch up with their harvest with that strategy (Figure 3). Team deep_greens had the highest total dosage, which did not, however, lead to high concentrations, due to an unfavorable ventilation strategy (data not shown).

**Figure 10:**
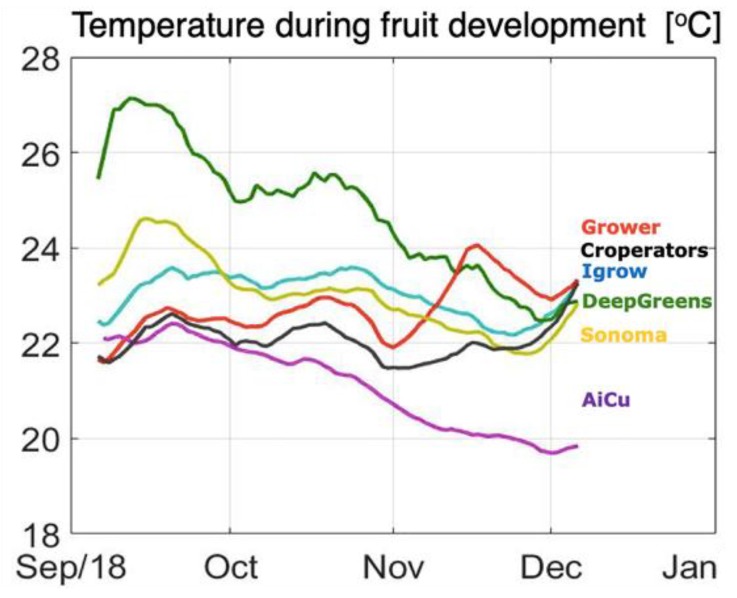
Course of average greenhouse air temperature during the fruit growth period in different compartments during the cucumber experiment. The lines are, therefore, the moving average of greenhouse air temperature in the fruit growth period preceding each harvest. The curve of AiCU, being a moving average of the temperature in 22 days, is therefore smoother than the curve of deep_greens, which is a result of a 13-day moving average.

Fruit growth duration is the time between flowering and harvest of a cucumber fruit, and varied between as little as 11 days to as much as 24 days. The overall average fruit growth duration was 17.3 days, but varied notably between the different teams. Deep_greens had the shortest (13.4 days) and AiCU the longest (21.6 days) average fruit growth duration. This correlates with the average greenhouse temperature during the fruit growth period (Figure 10). Figure 10 shows the average greenhouse temperature to which fruits were exposed during growth.

**Figure 11:**
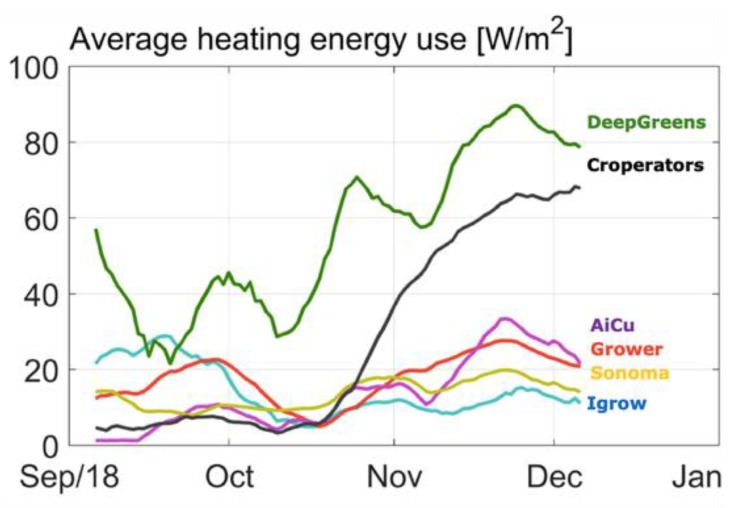
Course of heating energy use in different compartments during the cucumber experiment.

Figure 11 shows the amount of heating energy. Team deep_greens show a very different strategy from the others because their algorithm decided to create a very warm air temperature, probably to shorten the fruit development time. This resulted in a high resource use for heating (Table 1). Together with high daily light sums, mainly from artificial light (Figure 5b), while blocking natural light (Figure 5a), especially at the beginning, they were able to have a good harvest during the first weeks, but at the cost of high resource use on electricity (Table 1). Unfortunately, in October, technical problems (connection of AI remote control) and extremely low irrigation during several days led to a dip in harvest, from which they were not able to catch-up again.

Team AiCU applied relatively low temperatures (Figure 10) and a low daily light integral (Figure 4), while maintaining the highest fruit density (number/m^2^), which together explain the extremely high fruit abortion rate (Figure 7) and low light use efficiencies (Figure 6). The Croperators, due to the detection of small fungal disease spots in their compartment in November, were advised to lower the humidity levels. In order to reduce relative air humidity, they deactivated the misting system (which was intensively used in the second-half of October; data not shown) and started ventilating by opening both lee- and wind-side vents. Setting the minimum temperature of the pipe rail system to 40 °C allowed for maintenance of the air greenhouse temperature (night = 19 °C and day = 25 °C) and prevented it from falling, while ventilating. For this reason, the strategy led to a steep increase of the heating energy demand (Figure 11) but not to such an evident increase in the air temperature in the same timeframe.

**Figure 12:**
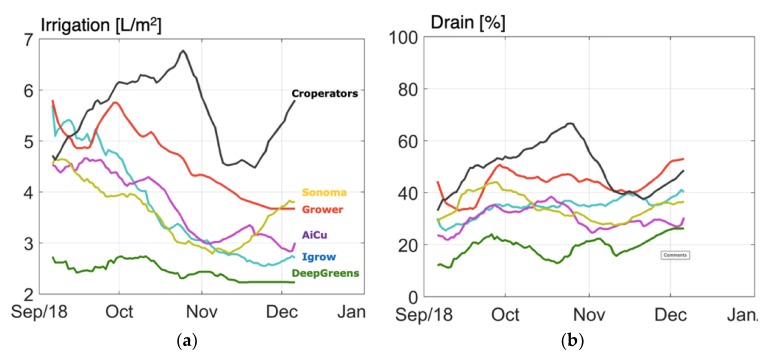
Course of irrigation water supply (**a**) and drain percentage (**b**) in different compartments during the cucumber experiment.

In Figure 12 the course of irrigation supply is given. Notable is the relatively high amount of irrigation supply of team The Croperators, and also the relatively high supply of the reference growers. In this experiment, high drain did not result in high water use because drain water was captured and fed back to the irrigation water, while taking the nutrients from the drain water into account when refilling the irrigation water buffer tank. High or low drain might affect the root quality, since it influences the oxygen availability. However, in this experiment such effects were not analyzed in detail.

In Table 1 the sustainability factors obtained during the growing experiment are presented for each team. In general, team Sonoma was able to realize the lowest resource use for CO_2_, heating, and water per kg cucumbers produced (class A + class B). Only on electricity use for artificial light did they realize average values. The reference team of growers obtained the lowest usage of electricity. In total, deep_greens obtained the highest resource use for heating, electricity, and CO_2_ (Table 1), which together with low production also led to a low net profit (Table 2). In Table 2, costs, income, and net profit are shown for each team. Highest net profit was obtained by team Sonoma, whose AI strategy resulted in a better performance than the manual growers. Sonoma won the challenge.

**Figure A5:**
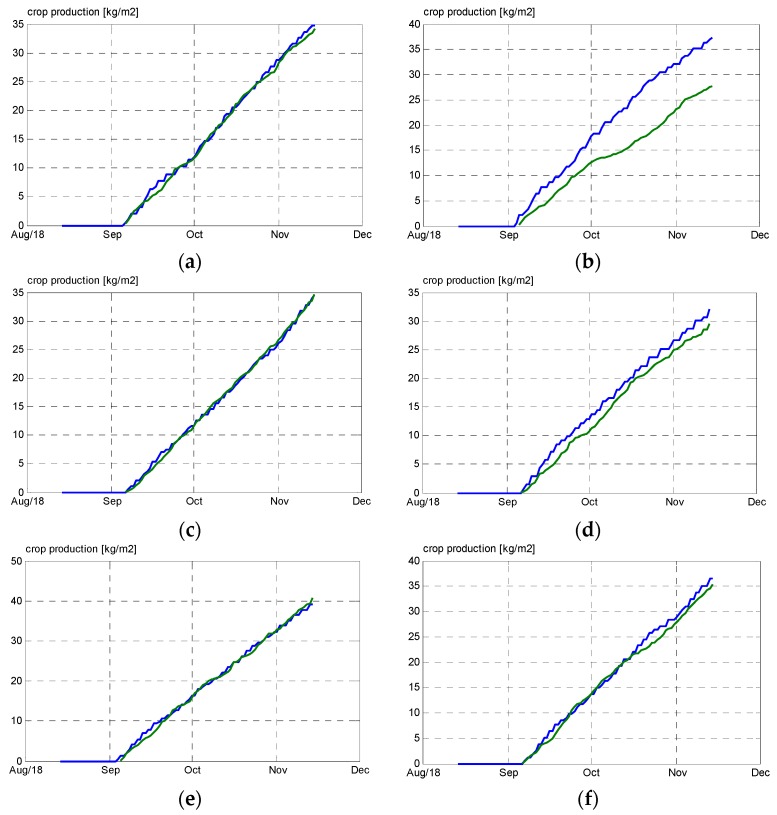
Validation results of modeled and realized crop production using realized climate setpoints and crop management strategies as input for model simulation for 5 teams: (**a**) iGrow, (**b**) deep_greens, (**c**) Grower = reference, (**d**) AiCU, (**e**) Sonoma, (**f**) The Croperators.

In order to analyze the different cropping, lighting, and climate control strategies, we used a combination of a dynamic climate and crop model. The validation of model predicted yield per greenhouse compartment and realized yield per team, and thus the greenhouse compartment is shown in Figure A5. A good agreement was found for all teams and compartments, except for deep_greens. The reasons for the poor agreement of predicted and realized yield for deep_greens were technical problems (connection of AI remote control) and extremely low irrigation over several days, which was not simulated by the combined model. The data of deep_greens is, therefore, not considered further in the analysis.

**Figure 13:**
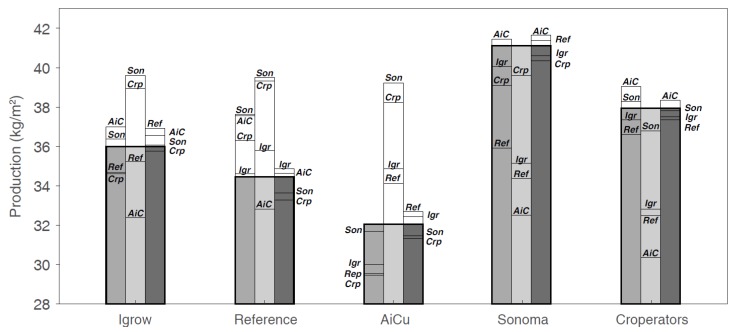
Realized cucumber production (greyscale bars) and predicted cucumber production per greenhouse compartment of different teams (iGrow, Reference, AiCU, Sonoma, The Croperators), using the cropping (1st bar), lighting (2nd bar), or CO_2_ (3rd bar) strategy of each of the other teams. Solid lines within the greyscale bars indicate lower production than that realized by the team, whereas the top colorless bars represent higher predicted production, e.g., team iGrow realized a cucumber yield of approximately 36 kg/m^2^; with the lighting strategy of Sonoma (but same climate and cropping strategy) they could have realized a yield of approximately 40 kg/m^2^, while with the lighting strategy of AiCU, it would have been only approximately 33 kg/m^2^.

**Figure 14:**
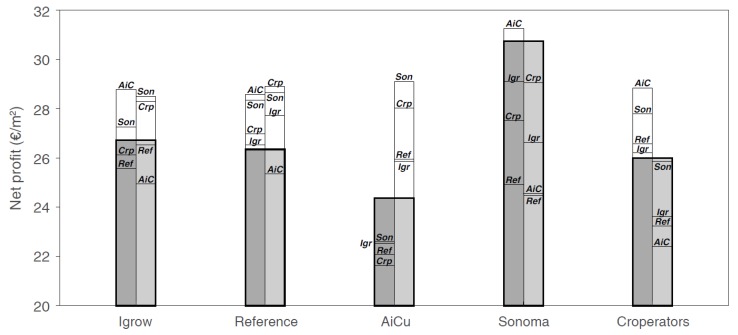
Realized net profit (greyscale bars) and predicted net profit per greenhouse compartment of different teams (iGrow, Reference, AiCU, Sonoma, The Croperators) using the cropping (1st bar), or lighting (2nd bar) strategy of each of the other teams. The predicted net profit under the different CO_2_ strategies for each team is not illustrated due to the limited effect on net profit.

In Figure 13 and Figure 14, the results of the strategy analysis on cucumber yield and net profit are shown, respectively. The winning team, Sonoma, could have improved their yield by applying the cropping and climate (temperature and CO_2_) strategy of AiCU, whereas AiCU could have improved their yield, and thus net profit, by applying the lighting strategy of Sonoma. From that we conclude that a dense crop with a high number of fruits per m^2^ is only effective when combined with a high light integral and high levels of temperature and CO_2_.

In summary, most teams were able to obtain a good production and low resource use and reach a net profit close to the performance of manual growers, or even better. All data of the growing experiment is published under doi 10.4121/uuid:e4987a7b-04dd-4c89-9b18-883aad30ba9a (Supplementary Materials).

## 4. Discussion

### 4.1. Crop Growing Strategy

The goal of climate and crop management is to maximize total crop growth rate by finding the best balance between climate factors (light, CO_2_, temperature) and crop characteristics (plant and stem density, leaf area, fruit removal), such that the maximum fraction of assimilates is distributed to the fruits. This is achieved by maintaining the maximum number of fruits per m^2^ without affecting photosynthetic capacity. If fruit load is too high in relation to photosynthetic capacity, young fruits will abort, which will have a negative impact on total production. If fruit load is too low, production will be relatively low, as there is a limit to fruit weight. The consequence is that there is a close association between the total number of fruits harvested and total fresh production. Cucumber fruits are harvested when they have achieved a certain weight, and fruit weight is closely related to fruit length [48], which is the most important quality trait.

Light is the basis for plant growth, as it provides the energy for photosynthesis [47,49]. CO_2_ air concentration is the other important environmental factor, being the carbon source for plant growth. Light and CO_2_ interact in a non-linear manner, but on the whole, both factors have a stronger positive effect at higher levels of the other factor [50,51]. Under the circumstances of the experiment, higher light integrals resulted in higher yields for the teams. Team iGrow, AiCU, and the Reference would have reached higher yields under the supplemental lighting use of Sonoma or The Croperators (Figure 13). The large relative effect of light on yield is illustrated by the fact that team Sonoma could have reached only slightly higher yields at higher CO_2_ dosages. Inter-changing CO_2_ application strategies did not result in strong production increase (Figure 13). This does not mean, however, that other settings with different levels of light and CO_2_ in combination with a different crop management could not have resulted in even higher productions. Furthermore, light and CO_2_ management are an integral part of greenhouse and crop management [52]. The scenarios illustrate the importance of simultaneously defining the lighting regime with crop management, plant and stem density, and leaf pruning strategy, as these affect light interception.

As photosynthetic capacity is mainly determined by light and CO_2_, teams that maximized these factors achieved the highest production (Figure 13). The difficulty is in anticipating future weather conditions. For example, if high light levels are expected, fruit load can be increased; however, if light levels remain low relative to the fruit load, abortion and un-even distribution of fruits over the stem will be the result. Growers manage their crops with this understanding, but may reduce risks by maintaining sub-optimal amounts of fruits in relation to future conditions. AI control can make such choices more explicit and find better combinations of growing conditions (combining weather forecasts, lighting, and CO_2_ strategies). This was demonstrated in this experiment, where the reference team of growers would have increased their yield and net profit by applying the lighting and cropping strategy of Sonoma.

The different lighting strategies of the teams illustrated the predominant effect of daily light integrals on yield and net profit. Among the factors affecting the economic feasibility of implementing higher light integrals are the energy costs and the product market price. Considering the low cost of electricity and the rule of thumb that a 1% increment in light returns a 1% increase in yield [50], the strategy of Sonoma to maximize the amount of artificial light while optimizing limiting factors, such as temperature and CO_2_, paid off.

### 4.2. Control Strategy

Greenhouse production is the result of complex interactions between physical, chemical, and biological processes of climate, water and nutrient supply, crop growth, and development [53]. Despite occurring instantaneously, the effects of climate on the crop are not immediately perceptible, but in response times that differ from less than a second (e.g., photosynthesis) to weeks (e.g., harvest weight). Crop responses to greenhouse management in conventional greenhouse systems are observed and evaluated by the growers who optimize the dynamic behavior of the system based on long-term accumulated experience and intuition [53]. In modern greenhouses, climate computers allow adjustment of a great amount of settings by the grower. However, crop management (fruit and leaf pruning, harvest) is decided upon, and carried out, manually. In the autonomous challenge, the climate in the manually grown reference was managed by growers with the aid of a climate computer that provided information on the outdoor and indoor climate. As modern greenhouse climate computers are parameterized in order to perform large parts of the greenhouse climate control automatically, the manually operated greenhouse required only occasional adjustments of the settings based on the observed and registered crop responses.

Optimal control refers to a control strategy that maximizes an explicit goal function [53]. Dynamic modelling is a key element towards intelligent determination of set points while considering optimal control. Previous research on this topic encompassed combined dynamic crop [18] and climate models [16] to determine temperature strategies that reduce production fluctuations and heat consumption in sweet pepper [19]. A multi-objective hierarchical control system was applied in a previous study [20] to determine reference trajectories for diurnal and nocturnal climate (temperature) and fertigation (EC) set points to maximize profit, tomato fruit quality, and water use efficiency. Mathematical dynamic models are built on scientific knowledge that describes the complex interactions within a greenhouse system. They enable a quantitative approach of the greenhouse system as transparent mechanistic models [13], allowing for optimization algorithms to find an optimal control, and they are physically interpretable.

Together with the expansion of cloud computing technology and the greater capacity of data, AI techniques are likely to be more suitable than mathematical models [54] in dealing with complex systems such as greenhouses. Continuous learning and adaptation of AI algorithms on historical data allows optimization of greenhouse processes and performance of certain tasks [45], such as determination of climate set points for growers. Previous studies in the field of greenhouse production on the control of climate variables with AI techniques have been carried out [21,22,28,35,54,55]. However, the studies were focused on the optimization of restricted amounts of environmental or crop variables.

During the autonomous greenhouse challenge, 5 teams developed an AI framework to reach a high net profit while maximizing the resource use efficiency of cucumber greenhouse production. The value of estimating optimal strategies on time series input depends on the future states of the system, which can be difficult to model using conventional supervised learning methods. Even though time series forecasting remains a complex task, deep neural networks have demonstrated in previous studies their ability to learn the non-linear dependencies and generate robust algorithms [56,57]. The complexity of the greenhouse production systems in these experiments was addressed by almost all teams with deep neural networks (deep_greens), architectures such as convolutional neural network (CNN; The Croperators) and recurrent neural network (RNN; AiCU tested this next to dynamic regression model and expert decisions), combined with reinforcement learning principles, such as model-based Bayesian reinforcement learning (BRL; Sonoma) and deep deterministic policy gradient( DDPG; iGrow used this together with GAN networks).

Model-based Bayesian reinforcement learning (BRL) has generated significant interest by the AI community. Learning with BRL is achieved by calculating the posterior distribution of the observed data. Incorporation of domain knowledge in prior distribution and notions of risks into the algorithm enables the acceleration of the learning process, yielding more robust policies [58,59]. BRL provides an elegant solution to the action-selection (exploration-exploitation) tradeoff in classical reinforcement learning [60]. Team Sonoma built a BRL algorithm that included different components aiming at encoding the expert policy and transferring it into a learnable model by means of imitation learning. The initialized agent operated on a continual model-based policy optimization process, that improved its performance through every environmental interaction. Due to the relatively short timeframe of the challenge, the team relied on a hand-crafted expert policy without accounting for continuous learning. Sonoma tried to incorporate the best knowledge on climate and crop management, starting from the assumption that light is a defining growth factor for a high-wire cucumber crop [50]. Based on this assumption they developed a temperature and CO_2_-based artificial light control optimization strategy. The algorithm accounted for short-term forecasts of outside global radiation to facilitate decisions on the control of the lighting system.

Deep Deterministic Policy Gradient (DDPG) is a policy-based learning algorithm, suitable for solving continuous actions, in which the agent directly learns from unprocessed observations through the policy gradient method, which relies upon the optimization of parametrized policies with respect to an expected return through gradient ascent [61]. Furthermore, Generative Adversarial Networks (GAN) belongs to a set of generative models, as they are able to produce new content. The GAN framework simultaneously trains a generative model that captures the distribution of data and a discriminative model that estimates the probability that a sample comes from training data rather than from the data of the generative model. The learning of the generative model is based on maximizing the probability of the discriminative model to detect the origin of the data. This competition enables improvement of both methods until the data origin is indistinguishable [62]. Team iGrow implemented a DDPG lifelong learning algorithm and a GAN framework to initially train their AI agent on a simulation environment and then transfer the knowledge gain into the greenhouse experiment. In their methodology their algorithm learned conditional distributions over future states of the greenhouse system, given the current states and possible actions that the agent could receive as an input. The agent queried the model with the various actions and selected those that maximized net profit by considering predictions of the cucumber price and resource efficiency. The final decisions were made by selecting the actions that resulted in less variance of the Q value.

The Croperators developed a greenhouse control system aiming to be easily implementable in commercial greenhouses. The conceptual scheme is based on a 3-layer approach trying to mimic the levels of greenhouse management normally performed by growers. The system ranges from a top layer that is close to the growers and includes a transparent understandable model, to a bottom layer that is based on black box CNN model. A similar hierarchical approach decision system has been previously described [54]. The top layer was used to determine the long-term crop management strategy based on a crop growth simulation model that is able to predict crop growth and the energy-climate requirements. The output of the top layer is the average climate targets of temperature, relative air humidity, CO_2_, and supplemental light needed to reach their goal function, which was maximizing net profit. The intermediate layer received the daily climate targets together with weather forecast, real time indoor climate, and data from the additional sensors of the team, and generated as an output the ideal climate trajectories, aiming at meeting the daily targets. The bottom layer was the actual operative section, meant to define the setpoints in order to reach the optimal climate profiler. Unlike the other teams The Croperators measured crop fresh weight with a weighing gutter, estimated fruit weight, and incorporated these data along with 24 h climate data into their algorithm. Data from the plant-based monitoring system of a previous study [28] were also included, as suggested in literature by different authors [28,54,63,64].

The teams integrated expert policy into their AI approach in a unified framework. Decisions regarding the climate and irrigation control strategies were taken autonomously in different time intervals by the learning algorithms, whereas the additional expert agents corrected for extremes. Despite the available results on crop responses, crop-related decisions, such as leaf pruning strategy for all teams, except The Croperators, were determined by expert policies and not by the algorithms.

Artificial datasets were initially generated with the available climate and crop models and enabled the development of the elementary framework. The predictive and prescriptive power of the algorithms depended partly on the training datasets, and may have restricted algorithms in making early-season crop management decisions. Algorithms capture information present in the data, and the limited variation among the attributes in the training datasets may have affected the performance of the algorithms in the beginning of the experimental period [65]. However, the idea of the experiment was that over the course of the season, the AI algorithms would be improved on the basis of provided management decisions and their effects on greenhouse climate and crop production. As could be observed, towards the end of the growing period, all algorithms indeed managed to establish a favorable greenhouse climate.

### 4.3. Sensors

Next to the standard sensors, teams used additional sensors (data not shown). We did not see a correlation of overall yield and net profit results obtained by the teams with the type and number of additional sensors used. Team Sonoma only used an additional leaf wetness sensor for their AI algorithm and obtained the overall best results. The good result was, however, satisfactorily explained by the high light integral and CO_2_ concentration. At the beginning of the experiment they also installed 8 RGB cameras. However, the images from these cameras were not used in the AI algorithm. Team iGrow did not install any additional sensors but was able to come close to performance of the reference. The Croperators installed the highest number of additional sensors. They installed several crop related sensors, such as a weighing gutter to determine crop transpiration (by measuring crop and substrate weight), and other sensors to monitor crop temperature, stem diameter, and crop sap flow meters. All the additional information from the other sensors did not seem to give them an obvious advantage concerning the end result. Therefore, no clear correlation of overall performance of teams can be made with the type and number of additional sensors used. However, since in this experiment important crop performance information was still obtained by manual counting and measuring, we cannot conclude that additional sensors may not be useful in the future. It might be that AI control of crops in greenhouses can substantially be improved by a dedicated choice of robust sensors giving direct information on crop performance, instead of indirect information on aerial or root zone environment of the crop (climate and irrigation). Automated information on crop growth parameters (stem elongation, fruit development time, leaves formation rate, cumulative number of leaves), now manually obtained by the greenhouse workers, would be essential.

## 5. Conclusions

The first successful benchmark experiment on remote control of greenhouse cucumber production with the use of state-of-the-art artificial intelligence algorithms has been carried out. We showed that AI algorithms can compete with experienced manual growers and can even outperform them.

However, more developments towards robustness, scalability, and generalization of algorithms will be essential. For the development of new AI algorithms, a large and complete set of training data with a wide range of variations will be needed. We have shown that artificial training data do provide a first step when real training data is lacking. However, current artificial data do not cover all aspects (e.g., pest and diseases) and provide only a limited description of the actual crop management.

To make the step towards a real “autonomous greenhouse”, it would be required to automate crop registrations and obtain automated data on pest and diseases. Next to that, it might be helpful to explore how manual labor in the greenhouses can be automated or robotized.

In the meantime, AI has affirmed that a combination of high amount of light, temperature, and CO_2_ at the right moments during the production process are essential to obtain high profits in an autumn cucumber crop cycle. Analyses showed that this strategy would have paid off even more at high plant densities. Manual growers can apply the lessons learned directly to their daily practice, even without AI.

AI-assisted or -managed greenhouse production can potentially improve crop production in locations where knowledge is limited, possibly also in greenhouses operated by highly skilled personnel. The technical make-up of the greenhouse has to meet certain minimum criteria to enable this. For example, sensor and communication facilities have to be in place, the greenhouse construction and installations should enable interventions, and fertigation and crop protections should meet minimum standards. Further development will be needed to make AI a full alternative for the top grower-skills that are nowadays required for (near)-optimal greenhouse production.

## Figures and Tables

**Table 1:** Sustainability factors of different teams obtained per kg cucumber (fresh weight) during the experiment.

	kg	kWh	kWh	L	mL
	CO_2_	Electricity	Heat	Water	Pesticide
per kg Cucumber					
Reference	0.20	3.02	3.20	5.52	0.34
Sonoma	0.20	3.59	2.49	4.91	0.35
iGrow	0.20	3.12	2.94	5.89	0.39
deep_greens	0.47	4.39	13.61	5.87	0.49
The Croperators	0.29	3.82	4.87	5.98	0.35
AiCU	0.26	3.17	3.13	7.62	0.48

**Table 2:** Costs and income of different teams per m^2^ greenhouse area.

	Reference	Sonoma	iGrow	Deep_Greens	The Croperators	AiCU
Young plants and substrate slabs	€3.74	€2.74	€3.74	€2.29	€2.74	€2.47
Electricity	€8.89	€10.97	€8.68	€9.35	€10.91	€7.04
Heating	€0.95	€0.77	€0.82	€2.92	€1.40	€0.70
CO_2_	€0.59	€0.62	€0.55	€1.00	€0.85	€0.59
Water	€0.27	€0.25	€0.28	€0.21	€0.29	€0.28
Labour	€8.32	€9.47	€8.85	€8.73	€9.48	€10.03
*Costs*	*€22.76*	*€24.82*	*€22.92*	*€24.50*	*€25.67*	*€21.11*
*Income*	*€43.94*	*€49.60*	*€42.95*	*€31.88*	*€42.82*	*€36.21*
*Net Profit*	*€21.18*	*€24.78*	*€20.03*	*€7.38*	*€17.15*	*€15.10*

## Supplementary Materials

Data collected in this research is available on https://doi.org/10.4121/uuid:e4987a7b-04dd-4c89-9b18-883aad30ba9a.
